# Proteolytic and antimicrobial activity of lactic acid bacteria grown in goat milk

**DOI:** 10.1080/13102818.2014.971487

**Published:** 2014-10-28

**Authors:** Jivka Atanasova, Penka Moncheva, Iskra Ivanova

**Affiliations:** ^a^ R&D Center, LB Bulgaricum PLC, Sofia, Bulgaria; ^b^ Department of General and Applied Microbiology, Biological Faculty, Sofia University ‘St. Kliment Ohridski’, Sofia, Bulgaria

**Keywords:** antimicrobial activity, goat milk, lactic acid bacteria, peptides, proteolytic activity

## Abstract

We examined 62 strains and 21 trade starter cultures from the collection of LB Bulgaricum PLC for proteolytic and antimicrobial activity of lactic acid bacteria (LAB) grown in goat milk. The aim of this study was to investigate the fermentation of caseins, α-lactalbumin and β-lactoglobulin by LAB, using the *o*-phthaldialdehyde (OPA) spectrophotometric assay and sodium dodecyl sulphate polyacrylamide gel electrophoresis (SDS-PAGE). The proteolysis targeted mainly caseins, especially β-casein. Whey proteins were proteolyzed, essentially β-lactoglobulin. The proteolytic activity of *Lactococcus lactis* l598, *Streptococcus thermophilus* t3D1, Dt1, *Lactobacillus lactis* 1043 and *L. delbrueckii* subsp. *bulgaricus* b38, b122 and b24 was notably high. The proteolysis process gave rise to medium-sized peptide populations. Most of the examined strains showed antimicrobial activity against some food pathogens, such as *Escherichia coli*, *Staphylococcus aureus*, *Salmonella cholere enteridis*, *Listeria monocytogenes*, *Listeria innocua* and *Enterobacter aerogenes*. The most active producers of antimicrobial-active peptides were strains of *L. delbrueckii* subsp. *bulgaricus* and *S. thermophilus*, which are of practical importance. The starter cultures containing the examined species showed high proteolytic and antimicrobial activity in skimmed goat milk. The greatest antimicrobial activity of the cultures was detected against *E. aerogenes*. The obtained results demonstrated the significant proteolytic potential of the examined strains in goat milk and their potential for application in the production of dairy products from goat's milk. The present results could be considered as the first data on the proteolytic capacity of strains and starter cultures in goat milk for the purposes of trade interest of LB Bulgaricum PLC.

## Introduction

Goat milk is known to have better digestibility, alkalinity and buffering capacity than cow's and human milk. Goat milk also has some therapeutic values in medicine and human nutrition. There has been much less research on goat milk than on the milk of other species, but distinct differences are becoming more known, revealing different processing requirements for yogurt and cheeses and for products related to human digestion and health.[1–3] For example, the relative proportions of the major casein components of goat milk are quite different from those in cow milk.[4] Goat milk contains less α_s_-casein, and often has more α_s2_ than α_s1_-casein. The latter is present in highly variable amounts depending on individual goats.[5] Proportions of κ-casein and especially β-casein are higher in goat milk than in cow milk. For an extensive review on the physico-chemical characteristics of goat milk, see [6].

To give a brief overview, casein contents in goat milk range from 16 to 26 g/L.[6] It is well known that the main whey proteins, α-lactalbumin (α-La) and β-lactoglobulin (β-Lg), are generally the major allergens in milk. In goat milk, the cDNA nucleotide sequence and the amino acid sequence of β-Lg has been described.[7] Two genetic variants of β-Lg have been reported in Spanish and French Saanen goats.[8] Low α-La percentages are the main characteristics of whey from different indigenous Greek sheep and goat breeds.[9] Moreover, the β-Lg percentage of goat acid whey is lower than that in sheep or cow acid whey. Also, the α-La in sheep and goat milk are closely homologous to cow α-La (reviewed in [6]).

Milk protein allergens can be degraded by some proteolytic enzymes produced by lactic acid bacteria (LAB) during microbial fermentation. That is why the fermentation with LAB is considered as an effective way to reduce whey proteins antigenicity.[10] Different strains of the genera *Lactobacillus*, *Streptococcus* and *Lactococcus* are used as starters in Bulgarian dairy products. In recent years, there is an increasing commercial interest in the production of bioactive peptides from milk, and especially from goat milk.

Bioactive proteins and peptides derived from milk are reported to provide a non-immune disease defence and control of microbial infections.[11] It is generally accepted that the total antibacterial effect in milk is greater than the sum of the individual contributions of immunoglobulin and non-immunoglobulin defence proteins such as lactoferrin, lactoperoxidase, lysozyme and peptides (reviewed in [12]). This may be due to the synergistic activity of naturally occurring proteins and peptides in addition to peptides generated from inactive protein precursors.[13] Milk proteins have also been proved to act as antimicrobial peptide precursors, and are thus speculated to enhance the organism's natural defences against invading pathogens.

The aim of this study was to evaluate the antimicrobial properties of LAB from different species and starter cultures after cultivation in goat milk, the proteolytic activity and the digestion of goat whey proteins.

## Materials and methods

### Microorganisms

Sixty-two LAB strains (16 strains of *Lactobacillus delbrueckii* subsp. *bulgaricus*: b24, Db, b120, b38, b122, b21, b208, b5, b8, b2, b41, b48, b144, b28, b7 and b2/1; 5 strains of *Lactobacillus helveticus*: h25, h9, h3P14, Q40 and h3; 1 strain of *Lactobacillus delbrueckii* subsp. *lactis*: 1043; 20 strains of *Streptococcus thermophilus*: t18, Dt1, t22, t8, t24, t26, t49, tn1, t37, t38, t2, t12, t17, Dt2, t4, t10, t39, t21 and t3D1; 21 strains of *Lactococcus lactis* subsp. *lactis*: l90, l598, l70, l216, l588, l400, l99, l9, l588/6, l310, l326/5, l47, l590, l526, l328, l247 and l160; and 2 strains of *Lactococcus lactis* subsp*. cremoris*: l603 and l162) and 21 starter cultures from the collection of LB Bulgaricum PLC, Sofia, Bulgaria (14 starter cultures for yoghurt: LBB BY 21, LBB BY 2, LBB BY 122-17, LBB BY 41-8, LBB BY 37-18, LBB BY D4, LBB BY 28-22, LBB BY 5-12, LBB BY 24, LBB BY 145-18, LBB BY 144-12, LBB BY 10, LBB BY 26 and LBB BY 145-18; 5 for white cheese: LBB C 1, LBB C 2, LBB C 3, LBB C 310-40 and LBB C 216-40; and 2 for cheese: LBB YC 1 and LBB YC 3) were used in this study.

For the determination of antimicrobial activity, the following test-microorganisms were used: *Escherichia coli* ATCC 11777, *Enterobacter aerogenes* ATCC 13048, *Staphylococcus aureus* ATCC 12600, *Salmonella choleraesuis* ser. *enteritidis* ATCC 13076, *Listeria monocytogenes* CECT 4032, *S. choleraesuis* ser. *thiphimurium* 123 (IBM), *Listeria inocua* F (ONIRIS, France), *Bacillus cereus* ATCC 11778 and *Bacillus subtilis* ATCC 6633.

### Nutrient media

The following culture nutrient media were used for the cultivation of the microorganisms: de Man–Rogosa–Sharpe (MRS) broth and agar (Merck), M17 broth and agar (Merck), Elliker broth and agar (Sharlau), nutrient broth, fresh skimmed goat milk and standard count agar medium (Merck).

### Antimicrobial activity

The antimicrobial activity of the LAB and the starter cultures was determined by the agar well diffusion method.[14] The cell-free supernatants obtained after the cultivation of the LAB in fresh skimmed goat milk and centrifugation at 3000 r/min for 30 min were used. In Petri plates with 20 mL of standard count agar medium, previously inoculated with 1.5 × 10^7^ cfu/mL (0.5 McFarland standards) of 24 h bacterial suspensions, wells were cut into the agar and filled with 20 μL of native and neutralized supernatants. After diffusion of supernatants at 4 °C for 2 h, the Petri plates were incubated at 37 °C for 24–48 h. The antimicrobial activity was assessed by measuring the diameter of the inhibition zone around the well. All experiments were performed in triplicate.

### Proteolytic activity

Strains were grown in MRS, M17 and Elliker broth. After that they were reinoculated from the liquid medium into MRS, M17 and Elliker agar. Collected cells were resuspended in 0.01 mmol/L K-phosphate buffer (pH 6.5) and inoculated in skimmed goat milk. The samples were incubated at 37 °C and 30 °C for 20 h. This method is based on the reaction of the free α-amino groups released by hydrolysis of casein with *o*-phthaldialdehyde (OPA) in the presence of β-mercaptoethanol to form a complex that strongly absorbs at 340 nm. The absorbance of the OPA reagent with aliquot of the control (non-inoculated skimmed goat milk) was substracted from each reading. The results were expressed in mmol/L of α-amino acid.[15]

### Polyacrylamide gel-electrophoresis

Analysis of the whey fractions obtained after cultivation of LAB strains and starter cultures was performed by Tris–Tricine polyacrylamide gel electrophoresis (PAGE), according to Shägger and van Jagow.[16] Tricine sodium dodecyl sulphate PAGE (SDS–PAGE) was carried out in 16.5% (w/v) polyacrylamide gels on vertical slab electrophoresis cells (BioRad Mini PROTEAN 3 System, Hercules, CA, USA) for 4 h at 60 V. The ultra low range molecular weight marker M3546 (MW 1060–26,600; Sigma-Aldrich) was used. Coomassie brilliant blue R250 was used to visualize the proteins.

## Results and discussion

### Antimicrobial activity

One of the selection criteria for probiotic strains is their ability to enhance natural defences of the host in respect of food pathogens by producing antimicrobial substances.[17]

The antimicrobial activity of 62 LAB strains and 21 starter cultures was investigated. For this purpose cell-free supernatants were used. To eliminate the inhibitory effect of the produced lactic acid, the pH of the supernatants was adjusted to 6.0. The active strains and starter cultures are shown in Table 1.

**Table 1.** Strains and starter cultures with antimicrobial activity.

	Test-microorganisms (active strains)					
	*E. coli*	*S. aureus*	*S. choleraesuis* ser. *enteritidis*	*S. choleraesuis* ser. *typhymurium*	*L. monocytogenes*	*E. aerogenes*
*L. delbrueckii* subsp*. bulgaricus*	(1) b24	(1) b144	(1) b5	(3) b7, b41, b48	0	(19) b144, b24, b38, b21, b5, b7, b2, b28, Db
*L. helveticus*						Q40, H25, h3P14, H9
*L. lactis*						1043
*S*. *thermophilus*		t2, t18, t12	t2		t2, t24	t2
Starter cultures	LBB BY 144-12, LBB BY 5-12	LBB BY 24, LBB BY 21, LBB BY 5-12, LBB BY 41-8		LBB BY 24, LBB BY 2, LBB BY 21, LBB BY 5-12, LBB BY 41-8, LBB C 216-40		

**Figure 1.**
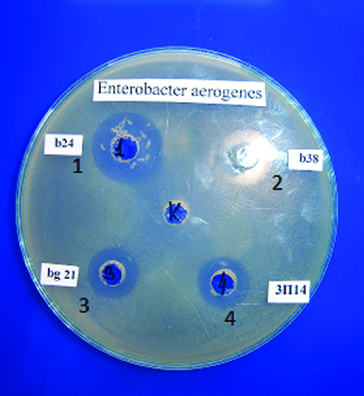
Antibacterial activity of selected LAB grown in goat milk against *E. aerogenes* ATCC 13048. b21, b24 and b38 – strains of *L. delbrueckii* subsp. *bulgaricus*; 3P14 (3П14) – *L. helveticus* strain.

In the present work, nine test-bacteria were used. Activity was not detected against *L. innocua* F, *B. subtilis* and *B. cereus*. The present results reveal that the strains belonging to the species *L. delbrueckii* subsp. *bulgaricus* had a relatively wide spectrum and were the most active ones against *E. aerogenes* (Figure 1). It could be noted that activity against *E. coli* was found for one strain of *L. delbrueckii* subsp. *bulgaricus* and for two starter cultures. *L. monocytogenes* was affected only by two *S. thermophilus* strains. It is interesting to note that *S. thermophilus* t2 had wider activity. All investigated lactococci were not active against any of the test-bacteria used. The main activity of the starter cultures was against *S. aureus* and *S. choleraesuis* ser. *typhymurium*. The data obtained confirmed the results of other authors [18–23] that the antimicrobial activity of LAB is variable and this is a general feature of LAB.

### Proteolytic activity

LAB utilize milk proteins as their prime source of essential and growth-stimulating amino acids. As many dairy starter cultures are highly proteolytic, bioactive peptides can be generated by starter and nonstarter bacteria used in the manufacturing of fermented dairy products. In addition to the use of live microorganisms, proteolytic enzymes isolated from LAB have also been successfully used to release bioactive peptides from milk proteins.[24]

**Figure 2.**
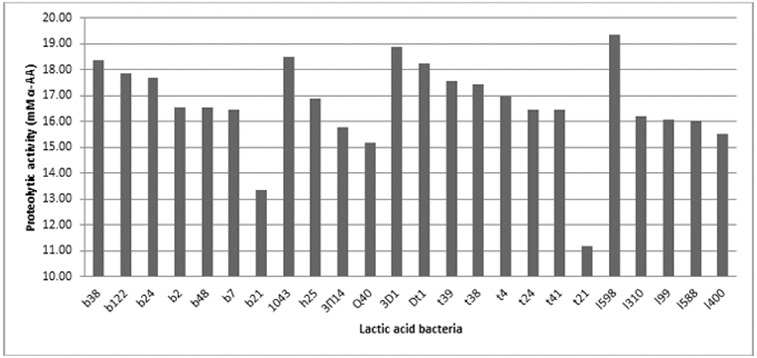
Proteolytic activity of selected LAB strains cultivated in goat milk. b38, b122, b24, b2, b48, b7 and b21 – strains of *L. delbrueckii* subsp. *bulgaricus*; 1043 – strain of *L. delbrueckii* subsp. *lactis*; h25, 3P14 (3П14) and Q40 – strains of *L. helveticus*; 3D1, Dt1, t39, t38, t4, t24, t41 and t21 – strains of *S. thermophilus*; 1598, 1310, 199, 1588 and 1400 – *L. lactis* strains.

The proteolytic activity of 58 of the LAB strains was investigated. It was shown that this activity varied among the strains, but not significantly (Figure 2). The proteolytic activity measured using the OPA method ranged between 10 and 20 mmol/L α-amino acids.

The proteolytic activity was higher for some strains of *L. lactic* subs. l*actis* (strain 1598), *S. thermophilus* (strains t3D1, Dt1, t39, t38), *L. delbrueckii* subsp. *lactis* (strains 1043) and *L. delbrueckii* subsp. *bulgaricus* (strains b38, b122 and b24) when grown in goat milk. The obtained results confirmed the published data on the proteolytic activity of LAB.[25] As LAB proteolytic systems are complex, there were several controversies regarding their ability to generate biopeptides. These peptides must be generated after the peptidase hydrolysis of long oligopeptides that are initially liberated through their proteinase activity during milk fermentation. As peptidase activity is intracellular in LAB, they probably contribute only after cell lysis, which is considered a rare event in fermented milk because of the short fermentation time. At the same time, LAB proteinases (*Lactococcus* sp. and *Lactobacillus* sp.) can hydrolyse more than 40% of the peptide bonds of α_S1_-CN and β-CN, resulting in the formation of more than 100 different oligopeptides that are then actively degraded by the complex peptidase system.[26]

### Digestion of goat whey proteins

Enzymatic hydrolysis of milk proteins can release fragments that are able to exert specific biological activities, such as antihypertensive, antimicrobial, opioid, antioxidant, immunomodulant or mineral binding.[6] Such protein fragments, known as bioactive peptides, are formed from the precursor inactive protein during gastrointestinal digestion and/or during food processing.[27] Due to their physiological and physico-chemical versatility, milk peptides are regarded as highly prominent components for health-promoting foods or pharmaceutical applications.[28]

**Figure 3.**
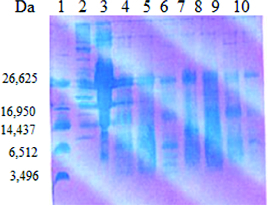
SDS-PAGE analysis of the whey fraction of goat milk after cultivation of selected LAB and starter cultures. Lane 1: ultra low molecular weight marker (Sigma-Aldrich); Lane 2: high molecular protein marker (Sigma-Aldrich); Lane 3: goat milk; Lane 4: starter culture LBB BY 21; Lane 5: *L. delbrueckii* subsp. *bulgaricus* b21; Lane 6: *S. thermophilus* t21; Lane 7: starter culture LBB YC-1; Lane 8: *L. helveticus* h3P14; Lane 9: *S. thermophilus* t3D1; Lane 10: *L. helveticus* h9.

**Figure 4.**
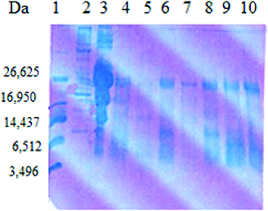
SDS-PAGE analysis of the whey fraction of goat milk after cultivation of selected LAB and starter cultures. Lane 1: ultra-low-molecular-weight marker (Sigma-Aldrich); Lane 2: high-molecular protein marker (Sigma-Aldrich); Lane 3: goat milk; Lane 4: starter culture LBB C-2; Lane 5: *L. delbrueckii* subs. *bulgaricus* b38; Lane 6: *S. thermophilus* t37; Lane 7: *L. helveticus* Q40; Lane 8: *S. thermophilus* tD1; Lane 9: starter culture LBB BY 145-18; Lane 10: *L. delbrueckii* subsp. *bulgaricus* b48.

The analysis of the whey fraction obtained after cultivation of the LAB strains and the starter cultures in goat milk was performed by Tris–Tricine PAGE (Figures 3 and 4). Hydrolysis of milk proteins resulted in differences in the distribution of peptides based on their molecular mass. This may be due to differences in proteinase–endopeptidase activity of the LAB.[29] The obtained results showed that low-molecular-weight protein fragments with molecular mass of 5–10 kDa were found after cultivation of the LAB strains and the starter cultures in skimmed goat milk. Such fragments were released by some strains belonging to the species *S. thermophilus* (t21, t3D1, tD1), *L. helveticus* (h9), *L. delbrueckii* subsp. *bulgaricus* b38 and starter cultures LBB BY21 and LBB BY 145-18. From these preliminary results, it could be speculated that the released low-molecular fragments from the digestion of whey proteins by some strains and starter cultures correlated with their antimicrobial activity (*L. delbrueckii* subsp. *bulgaricus* b38, *L. helveticus* h9 and LBB BY 21). Peptides derived from whey proteins have been reported to have antibacterial activity and attract more and more attention. These peptides showed a potent antimicrobial activity against a wide range of Gram-positive and Gram-negative bacteria.[30] El-Zahar et al. [31] found that the digestion of whey goat proteins as α-La and β-Lg with pepsin led to the production of peptides with activity against some food pathogens such as *E. coli* ATCC 11777, *B. subtillis* ATCC 6633 and *S. aureus* ATCC 12600 in a dose-dependent manner.

## Conclusions

The present work demonstrated the formation of peptides with molecular weight between 5 and 10 kDa confirmed with Tris–Tricine PAGE. The most active producers of antimicrobial-active peptides were strains of *L. delbrueckii* subsp. *bulgaricus* and *S. thermophilus*, which are of practical importance. The obtained results demonstrated the significant proteolytic potential of the examined strains in goat milk and their potential for application in the production of dairy products from goat's milk. To the best of our knowledge, this is the first one dealing with the antimicrobial and proteolytic activity of LAB strains and starter cultures from the collection of LB Bulgaricum PLC (Bulgaria) cultivated in goat milk.
